# Draft Genome Sequence of Aspergillus oryzae BP2-1, Isolated from Traditional Malted Rice in South Korea

**DOI:** 10.1128/MRA.01405-19

**Published:** 2020-01-02

**Authors:** Jongbum Jeon, Jung A Kim, Sook-Young Park, Gye-Won Kim, Cheon-Seok Park, Changmu Kim, Hye Yoon Park, Joo-Hong Yeo, Yong-Hwan Lee, Soonok Kim

**Affiliations:** a Department of Agricultural Biotechnology, Interdisciplinary Program in Agricultural Genomics, Center for Fungal Genetic Resources, and Center for Fungal Pathogenesis, Seoul National University, Seoul, Republic of Korea; b Genetic Resources Assessment Division, National Institute of Biological Resources, Incheon, Republic of Korea; c Department of Plant Medicine, Sunchon National University, Suncheon, Republic of Korea; d Brewing Research Center, Academic Industry Cooperation, Hankyong National University, Anseong, Republic of Korea; e Graduate School of Biotechnology, Kyung Hee University, Yongin, Republic of Korea; f Institute of Life Science and Resources, Kyung Hee University, Yongin, Republic of Korea; g Microorganism Resources Division, National Institute of Biological Resources, Incheon, Republic of Korea; Vanderbilt University

## Abstract

The fungus Aspergillus oryzae strain BP2-1 was isolated from the traditional malted starter culture nuruk. We report here the draft whole-genome sequence of A. oryzae BP2-1, which is comprised of 14 scaffolds with a total length of 39,455,382 bp and a GC content of 47.13%.

## ANNOUNCEMENT

Aspergillus oryzae is extensively used in industry, especially in the production of fermented foods and alcoholic beverages. Discovery of novel *A. oryzae* strains may assist in the development of novel enzymes and chemicals. An *A. oryzae* strain, BP2-1, which confers a unique flavor to makgeolli, was isolated during a survey of fungal strains from traditional nuruk in 2014. Moreover, extracts of malted rice with this strain exhibited lipid metabolism improvement activity by increasing peroxisome proliferator-activated receptor a (PPARa) activity in monkey kidney cells (CV-1) and a skin-lightening effect by inhibiting tyrosinase activity. Here, we report the draft genome sequence of *A. oryzae* BP2-1.

This strain was isolated from a nuruk sample collected from Donghae (Gangwon, South Korea) according to the protocols described by Yang et al. (1) and was deposited in the National Institute of Biological Resources (NIBR) culture collection under the accession no. NIBRFGC000134774. High-quality genomic DNA was extracted from *A. oryzae* BP2-1 germlings grown overnight at 23°C in potato dextrose broth with shaking at 120 rpm using a DNeasy minikit (Qiagen, Valencia, CA, USA) according to the manufacturer’s instructions. Genome sequencing of *A. oryzae* BP2-1 was conducted at Theragen Etex Bio Institute (Suwon, South Korea). One short-insert paired-end (PE) library (fragment size, 550 bp) and two long-insert mate pair (MP) libraries (insert sizes, 5 kb and 10 kb) were generated using a TruSeq DNA sample prep kit and a Nextera MP library prep kit (Illumina, CA, USA), respectively. The single-molecule real-time (SMRT) sequencing library was prepared according to the PacBio standard library preparation protocol using a PacBio DNA template prep kit (Pacific Biosciences, CA, USA), with a fragment size of 20 kb. A total of 996,216 reads with an average read size of 7,484 bp were generated on 8 cells of a PacBio RS II sequencer. Reads were assembled using an overlap-layout-consensus (OLC) algorithm and FALCON v. 0.2JASM (2), generating 41 contigs with a total length of 38,361,741 bp and an *N*_50_ value of 2,463,276 bp. After filtering with NextClip v. 1.3 (3), we obtained 46,143,916 reads with a Q30 of 88.70% from the PE library, 31,740,314 high-quality reads from the 5-kb inserted MP library, and 22,346,808 high-quality reads from the 10-kb inserted MP library from 101 cycles on an Illumina HiSeq 2000 platform. Using SOAPdenovo v. 2.04 (4), these short-read data sets were assembled into 78 contigs with a total length of 39,948,698 bp and an *N*_50_ value of 2,254,781 bp.

These two assemblies were merged into 23 contigs using HaploMerger2, followed by cleaning twice using the faDnaPolishing.pl script provided by HaploMerger2 (removeShortSeq=500) (5). Scaffolding and gap filling were performed using SSPACE-standard v3.0 (6), SSPACE-LongRead v1.1 (7), and GapFiller v1.10 (8), using default parameters. The final assembly consisted of 14 scaffolds with a total length of 39,455,382 bp and an *N*_50_ value of 4,366,015 bp. The GC content of the assembled genome was 47.13%. Genome assembly was validated using BUSCO v. 3.0.2b with the fungal ortholog data set (fungi_odb9) (9). In the assembly, 98.6% of the orthologs, including one that was duplicated, were found to be complete. This draft genome sequence will provide valuable information to identify genes for various bioactivities and will also facilitate comparative genomics with other publicly available *Aspergillus* fungal sequences for evolutionary studies.

### Data availability.

The genome sequence of Aspergillus oryzae BP2-1 obtained in this study has been deposited at GenBank under the accession no. NGZN00000000. The version described in this article is the first version, NGZN01000000. SRA data of PacBio and Illumina sequences were also deposited at GenBank under accession no. SRR9306672 to SRR9306682 (BioProject no. PRJNA368788).
